# Combined exercise training and dietary interventions versus independent effect of exercise on ectopic fat in individuals with overweight and obesity: a systematic review, meta‐analysis, and meta-regression

**DOI:** 10.1080/15502783.2025.2528534

**Published:** 2025-07-06

**Authors:** Fatemeh Kazeminasab, Motahareh Mohebinejad, Mohammad Hossein Mahboobi, Maedeh Nojoumi, Saba Belyani, Reza Bagheri, Fred Dutheil

**Affiliations:** a University of Kashan, Department of Physical Education and Sports Science, Faculty of Humanities, Kashan, Iran; b Mashhad University of Medical Sciences, Department of Nutrition, Faculty of Medicine, Mashhad, Iran; c The Ohio State University, Human Nutrition Program, Department of Human Sciences, Columbus, USA; d University of Isfahan, Department of Exercise Physiology, Isfahan, Iran; e University Hospital of Clermont-Ferrand, Preventive and Occupational Medicine, Witty Fit, Université Clermont Auvergne, CNRS, LaPSCo, Physiological and Psychosocial Stress, CHU Clermont-Ferrand, Clermont-Ferrand, France

**Keywords:** Exercise training, dietary intervention, metabolic health, metabolic disease, obesity

## Abstract

**Aim:**

While it is well established that reducing ectopic fat can help prevent insulin resistance in individuals with overweight or obesity, it remains unclear whether diet alone, exercise alone, or their combination is most effective in reducing specific ectopic fat depots. Therefore, the objective of this study was to investigate the effects of diet-only, and combined diet-plus-exercise interventions versus exercise only on ectopic fat reduction, and weight loss in adults with overweight or obesity.

**Methods:**

Web of Science, PubMed, and Scopus were searched for original articles, published until 1 March 2024 (no limitation on publication dates) that included diet only/or exercise and diet versus exercise alone on body weight, and ectopic fat in adults with overweight or obesity. Weighted mean differences (WMD) for body weight, liver fat, standardized mean differences (SMD) for visceral fat area (VFA), and intramuscular triglyceride (IMTG), and 95% confidence intervals (95% CI) were determined using random-effects models. Studies using noninvasive imaging techniques such as Computerized Tomography (CT), Magnetic Resonance Imaging (MRI), and hydrogen-based Magnetic Resonance Spectroscopy (H-MRs) for ectopic fat were included in this meta-analysis.

**Results:**

A total of 17 studies, including 732 participants aged 24.27 to 70.5 years (309 participants with metabolic diseases, and 423 without metabolic diseases) and 38 intervention groups, met the inclusion criteria. The combined intervention of exercise and diet significantly reduced body weight compared to exercise alone [WMD = -2.85 kg, *p* = 0.001], with significant reductions observed in both adults with and without metabolic disease, and for interventions lasting more than 12 weeks. However, the combined intervention did not significantly reduce liver fat, VFA, or IMTG compared to exercise alone. Diet-only interventions significantly reduced body weight compared to exercise alone [WMD = -2.57 kg, *p* = 0.010], but did not significantly affect liver fat, VFA, or IMTG. Meta-regression indicated that weight loss was a significant moderator of the effect of combined interventions on VFA (coefficient: −0.15; *p* = 0.030), but not for other outcomes. Also, based on subgroup analysis by intervention duration, both exercise and calorie restriction interventions in long-term (more than 12 weeks) have been successful in decreasing VFA in adults with overweight/or obesity.

**Conclusion:**

Combined exercise and dietary interventions are more effective than exercise alone in reducing body weight. While VFA was reduced following long-term interventions of exercise in combination with caloric restriction, our analyses showed no change in liver fat, or IMTG. Additional investigation is required to delve into the processes behind these findings and to pinpoint treatments that efficiently focus on reducing ectopic fat.

## 1. Introduction

Obesity represents a pathologically complex condition characterized by excessive adipose tissue deposition, with significant variations in its metabolic and clinical manifestations [1,2]. While dietary pattern modifications serve as the principal catalyst for rising obesity rates, concomitant reductions in physical activity levels act as important contributing elements in this epidemiological shift [3]. Ectopic fat refers to the pathological accumulation of triglycerides in non-adipose tissues that typically contain minimal fat stores, including the liver, pancreas, skeletal muscle, and myocardium [4]. Once an individual’s subcutaneous adipose tissue reaches its limit for expansion, it loses its ability to act as a protective metabolic reservoir [5,6]. Unlike subcutaneous fat, which is stored under the skin and is generally considered metabolically benign, and visceral adipose tissue (VAT), which is stored within the abdominal cavity around internal organs and has strong links to metabolic dysfunction, ectopic fat is stored within non-adipose tissues themselves. This shift in fat storage contributes to a higher burden on organs that were not originally designed for fat storage, leading to cellular dysfunction and metabolic disturbances. Chronic overnutrition initially promotes fat storage in subcutaneous adipose tissue, leading to adipocyte hyperplasia and/or hypertrophy. However, once these cells reach their expansion limit, lipid overflow occurs, redirecting free fatty acids to ectopic sites such as visceral organs [7]. This ectopic deposition, particularly in the liver (leading to nonalcoholic fatty liver disease [NAFLD]), skeletal muscle (linked to insulin resistance), and heart (associated with cardiomyopathy), plays a central role in the development of cardiometabolic diseases, and is considered more harmful than subcutaneous or even visceral fat in some contexts. Thus, differentiating between these fat depots is critical for understanding metabolic risk and tailoring effective interventions.

Intramuscular triacylglycerol (IMTG) is a form of triacylglycerol that is present in skeletal muscle fibers in smaller quantities as well as in subcutaneous and visceral adipose tissue (VAT) [8]. IMTG has been shown to play a dual role in metabolic processes; while it can serve as a readily available energy source during exercise, excessive accumulation is linked to insulin resistance and muscle dysfunction. Therefore, reducing ectopic fat deposits is a suitable target for interventions to prevent cardiovascular and metabolic diseases [9].

In light of the restricted availability of drugs that particularly target ectopic fat, researchers have shifted their attention to study the effectiveness of lifestyle interventions. In this regard, weight loss via diet and exercise reduces VAT [10,11] and liver fat [12–14] but reductions of 3% of body weight are generally required for hepatic benefit, with greater weight loss (5–10%) producing superior benefits [15]. Nevertheless, maintaining these levels of body weight reduction is intrinsically challenging [16].

Frequent physical activity is often associated with decreases in abdominal and visceral adipose tissue (VAT) and improvements in overall body composition, irrespective of dietary habits [17–21]. Moderate and vigorous physical activity, in particular, can reduce intrahepatic triglycerides, ectopic lipid deposits (in organs like the liver, skeletal muscle, and pancreas), body weight, waist circumference, and blood pressure [22–25]. For example, a comparison between calorie-restricted dietary interventions and exercise interventions with matched energy deficits showed that participants in the exercise group experienced a reduction in visceral fat that was twice as large as that in the calorie-restricted group [26]. However, some studies suggest that the impact of physical activity alone on VAT may be less pronounced compared to calorie-restricted diets when energy expenditure is not matched [27]. Moreover, the optimal exercise dose required to achieve targeted reductions in ectopic fat remains uncertain [28], and higher intensities or longer durations of exercise may be required to achieve significant reductions [29,30]. While energy imbalance is a key factor in obesity, additional contributors such as hormonal dysregulation, genetics, and environmental factors also play important roles [31–35]. Consequently, combining a low-calorie diet with regular physical exercise is widely recognized to offer greater benefits for weight and fat loss than either strategy alone [10,27,36].

Healthcare practitioners and academics generally recommend combining dietary modifications with regular physical exercise for managing body weight. Simultaneously implementing both interventions complicates the determination of the isolated effects of individual factors on weight loss. Systematic reviews, either alone or in combination with meta-analyses, have shown that it remains uncertain whether the combination of exercise training and a low-calorie diet affects body weight, liver fat, VFA, and IMTG differently compared to an exercise regimen combined with a normal diet (without caloric restriction), where the overall caloric deficit is similar in both groups, in individuals with overweight and obesity [37–39]. Moreover, a substantial portion of intervention studies have directly compared diet-only versus exercise-only interventions [27,40]. While this was not the initial primary focus of many analyses, it remains a critically important comparison for clinical decision-making. Understanding the relative effectiveness of dietary restriction versus exercise alone has practical implications for tailoring interventions, especially when patients are only willing or able to commit to one approach [41]. Therefore, this study also places emphasis on examining the independent effects of diet-only and exercise-only interventions, as well as the combination of both, in order to provide a comprehensive assessment of strategies for reducing ectopic fat (liver fat, VFA as primary outcomes, and IMTG as a secondary outcome), body weight loss (body weight as a primary outcome), and improving metabolic health.

## 2. Methods

### 2.1. Trial registration

The present systematic review and meta-analysis was conducted the guidelines set by the Preferred Reporting Items for Systematic Reviews and Meta-Analyses (PRISMA) guidelines [42] and followed the additional guidance provided by the Cochrane Handbook of Systematic Reviews of Interventions [43]. Additionally, the study was registered in advance with the International prospective register of systematic reviews (PROSPERO; ID: CRD42024551473).

### 2.2. Search strategy

A comprehensive search was conducted across electronic databases including Scopus, PubMed, and Web of Science. Two reviewers independently identified published research articles until 1 March 2024 (no limitation on publication dates) using the following key words: (“type 2 diabetes” or “diabetes mellitus” or “diabetes mellitus, type 2” or “non-insulin-dependent diabetes*” or “type II diabetes*” or “overweight” or “obesity” or “obese” or “obes*” or “insulin resistance” or “HOMA-IR” or “homeostatic model assessment for insulin resistance” or “metabolic syndrome” AND “caloric restriction” or “weight loss” or “diet” or “dietary” or “low calorie diet” or “energy restricted diet” or “calorie restricted diet” or “very low-calorie diet” AND “exercise” or “training” or “exercise training” or “physical activity” or “athletes” or “aerobic exercise” or “aerobic training” or “endurance exercise” or “endurance training” or “resistance exercise” or “resistance training” or “strength exercise” or “strength exercise” or “combined exercise” or “combined training” or “concurrent exercise” or “concurrent training” or “sports” or “exercise therapy” or “lifestyle intervention” or “anaerobic training”). The following keywords (“fatty liver” or “hepatic lipid” or “hepatic fat” or “IHTG” or “IHL” or “intra hepatic lipid” or “intra hepatic fat” or “intra hepatic triglyceride” or “hepatic lipid content” or “hepatic fat content” or “hepatic fat fraction” or “hepatic lipid fraction” or “liver lipid content” or “liver fat content” or “hepatic fat accumulation” or “hepatic lipid accumulation”) were used for liver fat, keywords (“muscle fat” or “muscular fat” or “skeletal muscle fat” or “muscle lipid” or “muscular lipid” or “skeletal muscle lipid” or “intramyocellular fat” or “intramyocellular lipid” or “intramyocellular triglycerides” or “muscular triglycerides” or “muscle fat fraction” or “muscle lipid fraction” or “muscle lipid content” or “muscle fat content” or “IMCL” or “IMTG”) for intramuscular triglyceride, keywords (“VAT” or “visceral adipose tissue” or “visceral fat” or “abdominal adipose tissue” or “abdominal fat” or “ectopic fat” or “ectopic adipose tissue”) for visceral fat. The filters including human, English language, and journal were applied. In addition, a manual search of the reference lists of all included studies was performed on Google Scholar. To ensure that all eligible studies were included in the present meta-analysis study. The searches were conducted independently by two authors, and any disagreements were resolved by discussion with another researcher.

### 2.3. Eligibility criteria and study selection

**Figure 1.**
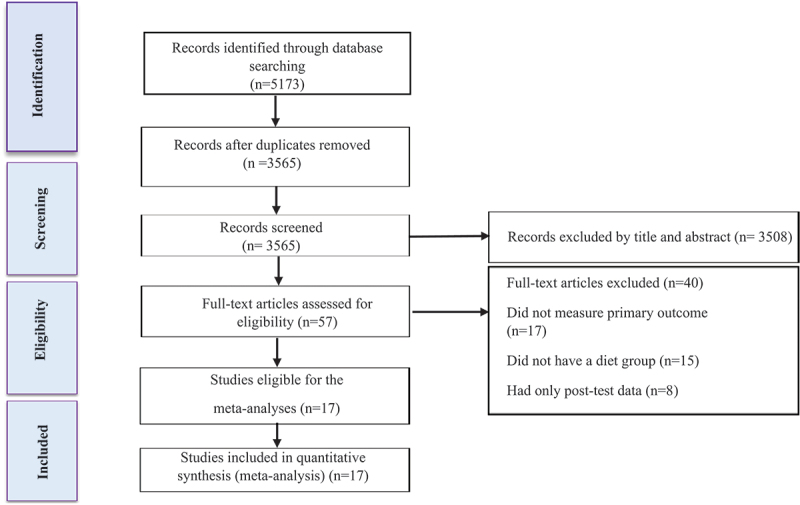
Flow diagram of systematic literature search.

The study selection process is shown in Figure 1. Articles were independently evaluated following removal of duplicate studies, titles and abstracts, in which full texts were reviewed by two reviewers to determine eligibility. Any disagreements were resolved through discussion with another author. The following study characteristics were extracted: (A) participant characteristics including health condition, biological sex, age, body mass index (BMI), and sample size; (B) exercise characteristics, and (C) diet characteristics, and duration of intervention (weeks). The participants with overweight (BMI ≥25 kg.m^2^), and obesity (BMI ≥30 kg.m^2^), were included in the present study. In addition, studies with an intervention duration of at least 3 weeks were included in the present study. Studies using noninvasive imaging techniques such as Computerized Tomography (CT), Magnetic Resonance Imaging (MRI), and hydrogen-based Magnetic Resonance Spectroscopy (H-MRs) for ectopic fat were included in this meta-analysis [44–47]. The current meta-analysis study focused on interventions based on diet, exercise or a combination of diet and exercise. For the current study, exercise was defined as any coordinated or supervised exercise program aiming to reduce body weight or body fat.

The data were extracted by two authors. For each outcome (body weight, liver fat, VFA, IMTG), pre- and post-intervention (means and standard deviations), or mean differences and associated standard deviations were entered into the meta-analyses to generate forest plots. If the means and standard deviations (SDs) were not reported, the SDs were calculated from standard errors of means (SEM) and medians and interquartile ranges (IQRs) [48–50].

### 2.4. Quality assessment and sensitivity analyses

Risk of bias was evaluated using the Physiotherapy Evidence Database (PEDro) scale [51]. We excluded two items (lack of blinding of participants and intervention providers) from the original 11-item scale because participants and intervention providers could not be blinded to the assigned diet conditions during studies. The scale used for the current study consisted of nine items: (1) specified eligibility criteria, (2) randomized participant allocation, (3) concealed allocation, (4) similarity of groups at baseline, (5) blinding of all assessors, (6) evaluated outcomes in 85% of participants, (7) intention-to-treat (ITT) analysis, (8) reporting of statistical comparisons between groups, (9) and point measures and measures of variability (Supplementary Table S1).

### 2.5. Statistical analysis

Meta-analyses were performed using the Comprehensive Meta-analysis (CMA) software (version 2.0, Biostat Inc., NJ, USA) to calculate weighted mean differences (WMD) or standardized mean differences (SMD) and 95% confidence intervals (CIs) for outcomes using random-effects models. Results were pooled using random-effects

models, based on the assumption that heterogeneity was likely from a clinical perspective, and may have affected the findings [52]. Effect sizes were calculated to compare the combined exercise training and dietary interventions versus independent effect of exercise on ectopic fat (body weight, liver fat, VFA, IMTG), or the impact of diet only compared to exercise only in individuals with overweight and obesity. Heterogeneity was evaluated by using the I^2^ statistic; significance was set at *p* < 0.05. According to Cochrane guidelines, I^2^ statistics were interpreted as follows: 25% as low, 50% as moderate, and 75% as high heterogeneity [53]. Subgroup analyses were performed according to health status (adults with or without metabolic diseases, intervention duration (short-term intervention ≤12 weeks, or long-term interventions > 12 weeks), and participant BMIs (adults with overweight (BMI ≥25 kg/m^2^, or obesity (BMI ≥30 kg/m^2^)).

Publication bias was detected through visual interpretation of funnel plots. If publication bias was present, Egger’s tests were used as a confirmatory test. Significant publication bias was deemed apparent if *p* < 0.1 [54]. Sensitivity analyses were also conducted for all outcomes using the “remove 1” technique. This procedure assessed whether individual studies had a disproportionate impact on the results of the meta-analyses [55]. Moreover, univariate meta-regression analyses of body weight and ectopic fat (visceral fat, and IMTG), or SFA were conducted comparing diet only vs. exercise alone, and combined exercise and diet versus exercise only.

## 3. Results

### 3.1. Included studies

Our initial search strategy identified 1244 articles from Scopus, 2428 articles from Web of Science, and 1501 articles from PubMed. After eliminating duplicate records (1608) and screening titles and abstracts (initial screening), 57 studies were retrieved for a more detailed appraisal of the full texts (secondary screening). Forty studies were excluded after reviewing full text for the following reasons: (A) 17 did not measure primary outcomes (liver fat, VFA, and IMTG), (B) 15did not have an exercise group, (C) eight studies had only posttest data. A total of 17 studies, inclusive of 38 intervention groups, was included in the present systematic review and meta-analysis. A flow diagram of the systematic literature search is presented in Figure 1.

### 3.2. Participant characteristics

A total of 732 participants was included with sample sizes ranging from 16 [40,56,57] to 109 [58]. The mean age of participants ranged from 24.27 [59] to 70.5 [58] years, and the mean body mass index (BMI) of participants ranged from 26.4 to 40 kg.m^−2^. The mean age of exercised and diet participants ranged from 44 [60] to 70.6 [61] years, the mean age of diet only groups ranged from 26.36 [59] to 66.9 [40] years, and the mean age of exercise only participants ranged from 44 [40] to 69.9 [58] years. The mean BMI of exercise and diet participant group ranged from 26.4 [62] to 33.7 [63] kg.m^−2^, the mean BMI of diet only groups ranged from 26.6 [62] to 34.3 [64] kg.m^−2^, and the mean BMI of exercise only groups ranged from 26.8 [65] to 37 [57,60] kg.m^−2^. Both males and females were included in 13 studies [40,56–63,65–68], females only in one study [64], and males only in three studies [27,69,70]. All participants were overweight/obese and with or without metabolic diseases (309 participants with metabolic diseases, and 423 without metabolic diseases). Table 1 presents the full details of participant characteristics.

**Table 1.** Characteristics of included studies.

Study	Sample size (sex)	Health condition	Groups	Outcomes	Measure	Age [years] Mean±SD (range)	BMI [kg.m^2^] Mean±SD	Exercise intervention	Follow up (week)	Diet intervention
Ard et al. [58]	109 (M&F)	Obese	Diet (CR-WM) Diet (CR-WL) A-Exe	VFA	MRI	Diet (CR-WM): 70.5 ± 4.8 Diet (CR-WL): 70.3 ± 4.8 A-Exe: 69.9 ± 4.5	Diet (CR-WM): 30–40 Diet (CR-WL): 30–40 A-Exe: 30–40	A-Exe included 90–150 min/week of moderate to vigorous cardio-aerobic exercise such as walking based on monitoring their heart rate	52	CR received recommendations for received diet advice: eat more low-energy dense fruits, veggies, lean protein, whole grains. Aim for 25% protein, 47% carbohydrate, 28% fat.
Cai et al. [59]	44 (M&F)	NAFLD Overweight	Diet (LCD) A-Exe	VFA LF	MRI and MRS	Diet (LCD): 25.36 ± 3.517 A-Exe: 24.27 ± 2.0167	Diet (LCD): 27.85 ± 3.517 A-Exe: 27.33 ± 3.236	A-Exe 30 minutes a day of moderate to intense exercise with a heart rate of 60 to 70 Maximum heart rate	3	LCD Carbohydrate consumption less than 50 grams per day without calorie restriction
Cheng et al. [62]	67 (M&F)	NAFLD Prediabetes	Diet (CR) A-Exe+Diet (CR) A-Exe	VFA LF	MRS	Diet (CR): 60 ± 4.1 A-Exe+Diet (CR): 60 ± 3.5 A-Exe: 59 ± 4.4	Diet (CR): 26.6 ± 2.7 A-Exe+Diet (CR): 26.4 ± 2.9 A-Exe: 27.3 ± 10.6	A-Exe At first 5 min warm-up and the end 5 min cool-down, Progressive supervised -Ex training (60–75% VO2max intensity) was given 2–3 times/week in 30–60 min/sessions.	48	CR meal included 37–40% carbohydrate with 9–13 g as fiber, 35–37% fat (SAFA 10%, MUFA 15–20%, PUFA 10%) and 25–27% protein..
Croci et al. [71]	16 (M&F)	NAFLD Obese	Diet (CR) A-Exe	VFA	MRI	Diet (CR): 51.8 ± 6.7 A-Exe: 45.5 ± 13.5	Diet (CR): 31.2 ± 3.2 A-Exe: 33.5 ± 9.0	A-Exe 3 sessions/week circuit training for 6 months. Intensity: 50% 1-RM. Volume increased from 1 to 5 circuits/week over 11 weeks, then constant. Program: 30s exercise, 30s rest intervals.	26	CR Diet plan: aim for 5%-10% body weight loss in 16 weeks, followed by 8 weeks focusing on body weight. Target macronutrients: 40% carbs, 20% protein, 40% fat ( < 10% saturated fat).
Dube et al. [40]	16 (M&F)	Obese	Diet (CR) A-Exe	IMTG	Biopsy and Oil Red O staining	Diet (CR): 66.9 ± 4.80 A-Exe: 68.4 ± 4.24	Diet (CR): 31.2 ± 3.39 A-Exe: 30 ± 2.82	A-Exe moderate (determined by heart rate or perceived exertion with cycling and some walking), 4–5 days/week, 45 min/session about (180 min/week)	16	CR The goal of diet intervention was to achieve a 10% weight loss; a reduction of 2,093.4–4,186.8 kJ/day (based on recent food records/history) and low fat ( < 30% of energy from fat) diet
Erikson et al. [57]	16 (M&F)	Obese	A-Exe+Diet (CR) A-Exe	VFA	CT scan	A-Exe+Diet (CR): 67.0 ± 5 A-Exe: 65.0 ± 4	A-Exe+Diet (CR): 35 ± 5 A-Exe: 37 ± 5	A-Exe 5 days/week, for 50–60 min in duration with 60–85% MHR on either a treadmill or cycle ergometer	12	CR Hypocaloric diet: reduce daily calories by 500 kcal
Ezpeleta et al. [60]	54 (M&F)	NAFLD Obese	Diet (ADF) A-Exe+Diet (ADF) A-Exe	LF VFA	LF: MRI-PDFF VFA: DXA,	Diet (ADF): 44.0 ± 13.41 A-Exe+Diet (ADF): 44.0 ± 13.41 A-Exe: 44.0 ± 13.41	Diet (ADF): 36 ± 34.87 A-Exe+Diet (ADF): 37 ± 22.36 A-Exe: 37 ± 26.83	A-Exe 5 days a week, for 60 minutes at 65–80% MHR on a treadmill, stationary cycle, or elliptical machine	12	ADF Subjects ate 600 kcal for dinner (5–8 pm) on fast days and ate freely on feast days starting at 12 am each day. They drank water and energy-free beverages like black coffee, herbal tea, black tea, and max 2 sugar-free sodas per day.
Giannopoulou et al. [64]	33) F)	Obese Type 2 diabetic Menopause	Diet (CR) A-Exe+Diet A-Exe	VFA	MRI	Diet (CR): 58.5 ± 5.637 A-Exe+Diet: 57.4 ± 5.637 A-Exe: 55.5 ± 5.637	Diet (CR): 34.3 ± 6.3 A-Exe+Diet: 33.7 ± 6.3 A-Exe: 35.9 ± 6.3	A-Exe Intervention consisted of a supervised walking program 3 to 4 times per week for 60 minutes at 65% to 70% V˙ o2 peak and approximately 1050 to 1250 kJ were expended per exercise session	14	CR Intervention consisted of a high-monounsaturated fat (HMF) diet composed of 40% fat (30% monounsaturated,5% polyunsaturated, and 5% saturated), 40% carbohydrates (15% simple and 25% complex carbohydrates), and 20% protein
Murphy et al. [72]	33 (M&F)	Overweight Postmenopausal	Diet (CR) A-Exe	IMTG VFA	IMTG: MRI VFA: MRI	Diet (CR): 55 ± 2.88 A-Exe: 59 ± 2.8	Diet (CR): 26.7 ± 2.06 A-Exe: 26.8 ± 2	A-Exe they increased energy expenditure by 16% in the first 3 months and by 20% in the following 9 months through cardio exercise.	52	CR Participants aimed to reduce calorie intake by 16% in the initial 3 months and by 20% in the subsequent 9 months in the CR intervention
Nicklas et al. [66]	101 (M&F)	Obese	R-Exe+Diet (CR) R-Exe	IMTG	IMTG: advantage windows 4.2 volume viewer	R-Exe+Diet (CR): 69.6 ± 3.9 R-Exe: 69.4 ± 3.2	R-Exe+Diet (CR): 30.4 ± 2.2 R-Exe: 30.7 ± 2.4	R-Exe 3 sets ×10 reps, 1 min rest between sets, 3 days/week at 70% 1RM. Machines: 1) leg press, 2) leg extension, 3) seated leg curl, 4) seated leg press, 5) incline press, 6) compound row, 7) triceps press, 8) biceps ring. Warm up: 5 min walk/cycle, light stretch, 5 min cool down/stretch.	20	CR Each participant received a daily caloric intake by deducting 600 kcal from their estimated energy needs for weight maintenance. They were given up to 2 meal replacements per day (shakes and bars; Slim-Fast Inc.) with approximately 220 kcal, 7–10 g protein, 33–46 g carbohydrates, 1.5–5 g fat, and 2–5 g fiber for breakfast and lunch. Dinner and snack choices were personalized by the RD based on caloric goals and individual food preferences.
Racette et al. [73]	38 (M&F)	Overweight postmenopausal	Diet (CR) A-Exe	VFA	VFA: MRI	Diet (CR): 55.6 ± 3.48 A-Exe 58.8 ± 2.61	Diet (CR): 27.2 ± 2.61 A-Exe: 27.2 ± 1.74	A-Exe Increase daily energy expenditure through exercise (including treadmills, tracks, cycle ergometers, rowing machines, elliptical machines, and stair climbers) without changing caloric intake. The Exe version started at 16% and went up to 20%.	52	CR Reduction of daily energy consumption up to 16% for the first 3 months of the intervention and 20% for the remaining 9 months
Ross et al. [27]	33 (M)	Obese	Diet (CR) Com-Exe	VFA	MRI	Diet (CR): 42.6 ± 9.7 Com-Exe: 46.0 ± 10.9	Diet (CR): 30.7 ± 1.9 Com-Exe: 30.7 ± 1.6	A-Exe 5 days a week 15 to 60 minutes R-Exe 3 days/week, 8 exercises and 8–12 repetitions with 30–45% of 1RM in each session	16	CR weight-maintenance energy intake was reduced by 4.19 MJ/day (1,000 kcal/day).
Santanasto et al. [61]	32 (M&F)	Obese	Com-Exe+Diet (CR) Com-Exe	IMTG VFA	IMTG: CT VFA: CT	Com-Exe+Diet: 70.6 ± 5.9 Com-Exe: 69.9 ± 5.9	Com-Exe+Diet: 33.6 ± 3.3 Com-Exe: 32.1 ± 3	Com-Exe Walking on a treadmill for at least 150 minutes per week as the primary mode of activity. To complete the walk, participants completed lower extremity resistance training, balance and stretching exercises.	26	CR Daily fat intake limited to 25% of total calories. Focus on monounsaturated and polyunsaturated fats, limit saturated fat and cholesterol. Include 5 servings of fruits or vegetables and 6 servings of whole grains daily
So et al. [69]	58 (M)	Overweight Obese	Diet (CR) A-Exe	VFA	Multiple-slice imaging protocol	Diet (CR): 46.6 ± 4.9 A-Exe: 47.6 ± 10.1	Diet (CR): 30.9 ± 4.6 A-Exe: 29.8 ± 4	A-Exe 3sesions/week,30–60 min of brisk walking and/or mild jogging performed outdoors 15–30 min of warm-up 15–30 min of cool-down	12	CR Consuming a balanced diet with 1680 kcal per day based on Four-Food-Group Point Method
Solomon et al. [74]	23 (M&F)	Obese	A-Exe+Diet (CR) A-Exe	IMTG	MRI	A-Exe+Diet: 67 ± 3.31 A-Exe: 66 ± 1.46	A-Exe+Diet: 33.5 ± 4.97 A-Exe: 34.7 ± 5.54	A-Exe moderate intensity aerobic exercise (treadmill walking/cycle ergometry/stationary rowing), 5 days/week with 75% VO2max, 60 min/session	12	CR Hypocaloric diet: reduce their daily energy intake about 500 cal
Thong et al. [70]	30 )M(	Obese	Diet (CR) A-Exe	VFA	MRI	Diet (CR): 42.6 ± 9.726 A-Exe: 45.0 ± 7.6	Diet (CR): 30.7 ± 1.870 A-Exe: 32.3 ± 2	A-Exe The exercise program involved daily supervised brisk walking or jogging on a treadmill for 12 weeks, with session length based on burning 700 kcal and intensity capped at 75% V˙ O2 max (;80% max heart rate).	12	CR maintenance diet by 700 kcal/day.
Yassine et al. [63]	24 (M&F)	Obese Metabolic syndrome	A-Exe+Diet (CR) A-Exe	VFA	CT scan	A-Exe+Diet (CR): 65.5 ± 5.0 A-Exe: 65.5 ± 5.0	A-Exe+Diet (CR): 33.7 ± 4.7 A-Exe: 35.3 ± 5.8	A-Exe Participants exercised 50–60 min/day, 5 days/week, for 12 weeks. They walked on a treadmill and/or pedaled a cycle ergometer, with over 75% effort on the treadmill. Initial sessions were at 60–65% HR max, gradually reaching 80–85% HR max (~70% VO2max) by Week 4.	12	CR In the Exe+CR group, participants were required to reduce their energy intake by 500 kcal/d

Abbreviations: M: Male; F: Female; CR: Calorie restriction; WM: Weight maintenance; WL: Weight loss; A-Exe: Aerobic exercise; R-Exe: Resistance exercise; Com-Exe: Combined exercise; VFA: Visceral fat area; MRI: Magnetic Resonance Imaging; NFLD: Non-alcoholic fatty liver disease; LCD: Low-carbohydrate diet; LF: Liver fat; MRS: Magnetic Resonance Spectroscopy; IMTG: Intramuscular triglyceride; ADF: Alternate-day fasting.

### 3.3. Intervention characteristics

Intervention durations ranged from three [59] to 52 [58,65,67] weeks, with 12-week durations in the majority of studies [57,60,63,68–70]. In one study, resistance exercise and diet was compared with an exercise alone [27], eight studies compared aerobic exercise and diet vs. exercise only and 11 studies compared diet only vs. exercise only [40,56,58–60,62,64,65,67,69,70], and one study compared combined exercise and diet vs. exercise only [61]. One study used more than one type of exercise protocol as separate interventions [27]. Exercise sessions were performed two [75] to five [27,40,57,60,63,68,71] times per week, with three sessions being the most common.

The intensity of each session of resistance training ranged from 30% one-repetition maximum (1RM) [27] to 70% 1RM [66]. The duration of each session of aerobic training ranged from 15 [27] to 60 [27,57,60,64,68,69] minutes. The intensity of each session of aerobic training ranged from 60% [57,59,63] to 85% [57,63] maximum heart rate (MHR). The duration of each session of combined exercise varied from 30 to 70 min. The characteristics of intervention are presented in Table 1.

### 3.4. Meta-analysis

#### 3.4.1. Diet only vs. exercise only

##### 3.4.1.1. Body weight

**Figure 2.**
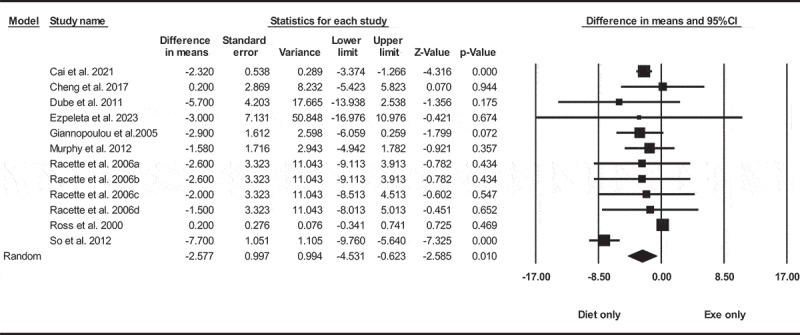
Forest plot of the effects of diet only vs. exercise only on body weight. Data are reported as WMD (kg) (95% confidence limits). WMD, weighted mean difference.

Based on 12 intervention arms with 431 participants, diet only significantly reduced in body weight [WMD = −2.57 kg (95% CI −4.53 to −0.62), *p* = 0.010], when compared with an exercise only group (Figure 2). There was significant heterogeneity among included studies (I^2^ = 83.92%, *p* = 0.001). Visual interpretation of funnel plots and Egger’s test (*p* = 0.120) results also did not show publication bias. Sensitivity analysis performed by removing individual studies revealed that removing the Cai et al. 2021 study resulted in a change in the effect size and significance (WMD = −2.61 kg, *p* = 0.050), while the direction of the results remained consistent.

Subgroup analyses by health status revealed diet only significantly reduced in body weight for adults with metabolic disease [WMD = −2.24 kg (95% CI −3.14 to −1.33), *p* = 0.001, nine interventions], but not for without metabolic disease [WMD = −4.16 kg (95% CI −10.75 to 2.41), *p* = 0.210, three interventions], when compared with an exercise only group.

In addition, subgroup analyses by intervention duration revealed diet only did not significantly reduce in body weight for long-term > 12 weeks [WMD = −0.22 kg (95% CI −0.97 to 0.52), *p* = 0.550, nine interventions], or short-term interventions ≤12 weeks [WMD = −4.74 kg (95% CI −9.58 to 0.09), *p* = 0.050, three interventions], when compared with an exercise only group.

In addition, subgroup analyses by BMI-indicated diet only significantly reduced in body weight for adults with overweight [WMD = −2.24 kg (95% CI −3.20 to −1.28), *p* = 0.001, six interventions], but not for individuals with obesity [WMD = −1.37 kg (95% CI −3.96 to 1.21), *p* = 0.290, four interventions], when compared with an exercise only group.

##### 3.4.1.2. Liver fat

**Figure 3.**
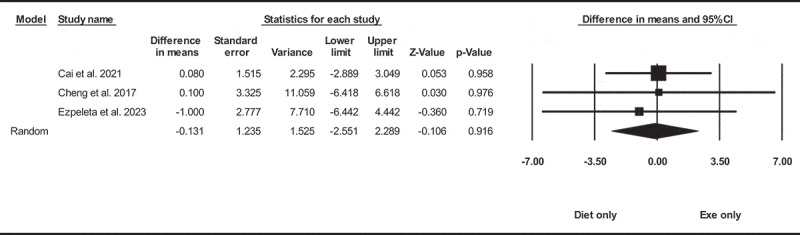
Forest plot of the effects of diet only vs. exercise only on liver fat. Data are reported as WMD (percent) (95% confidence limits). WMD, weighted mean difference.

Based on three intervention arms with 122 participants, diet only did not significantly change liver fat [WMD = −0.13% (95% CI −2.55 to 2.28), *p* = 0.910], when compared with an exercise only group (Figure 3). There was not significant heterogeneity among included studies (I^2^ = 0.00%, *p* = 0.940). Visual interpretation of funnel plots and Egger’s test (*p* = 0.650) results did not show publication bias. Sensitivity analysis performed by removing individual studies showed that the significance of the results and the direction of the results did not change.

Due to the small number of studies, it was not possible to perform subgroup analysis based on health status, intervention duration, or BMI.

##### 3.4.1.3. Visceral fat area (VFA)

**Figure 4.**
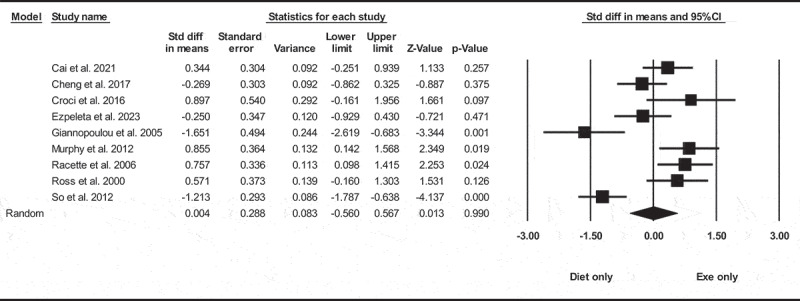
Forest plot of the effects of diet only vs. exercise only on VFA. Data are reported as SMD (95% confidence limits). SMD, standardized mean difference.

Based on nine intervention arms with 1487 participants, diet only did not significantly change VFA [SMD = −0.004 (95% CI −0.56 to 0.56), *p* = 0.990], when compared with an exercise only group (Figure 4). There was significant heterogeneity among included studies (I^2^ = 82.80%, *p* = 0.001). Visual interpretation of funnel plots and Egger’s test (*p* = 0.740) results also did not show publication bias.

Sensitivity analysis performed by removing individual studies showed that the significance of the results and the direction of the results did not change.

Subgroup analyses by health status revealed diet only did not significantly decrease in VFA for adults with metabolic disease [SMD = 0.11 (95% CI −0.45 to 0.06), *p* = 0.690, seven interventions], or without metabolic disease [SMD = −0.33 (95% CI −2.08 to 1.41), *p* = 0.700, two interventions], when compared with an exercise only group.

In addition, subgroup analyses by intervention duration revealed diet only did not significantly change in VFA for long-term > 12 weeks [SMD = 0.21 (95% CI −0.48 to 0.90), *p* = 0.540, six interventions], or short-term interventions ≤12 weeks [SMD = −0.37 (95% CI −1.31 to 0.55), *p* = 0.42, three interventions], when compared with an exercise only group.

In addition, subgroup analyses by BMI indicated diet only did not significantly change in VFA for adults with overweight [SMD = 0.17 (95% CI −0.80 to 1.15), *p* = 0.720, four interventions], or individuals with obesity [SMD = −0.40 (95% CI −1.55 to −0.74), *p* = 0.490, three interventions], when compared with an exercise only group.

Meta-regression determined whether or not body weight loss influenced the effects of diet only on VFA, in which no significant correlation was found (coefficient: −0.049; 95% CI, −0.28 to 0.18; *p* = 0.690). This result suggested that there was no significant moderating effect of body weight loss.

##### 3.4.1.4. Intramuscular triglyceride (IMTG)

Based on two intervention arms with 49 participants, diet only did not significantly change IMTG [SMD = −2.06 (95% CI −4.36 to 0.23), *p* = 0.070], when compared with an exercise only group (Supplementary Figure S1). There was significant heterogeneity among included studies (I^2^ = 86.76%, *p* = 0.006).

Due to the small number of studies, it is not possible to perform subgroup analysis based on health status, intervention duration, and BMI.

#### 3.4.2. Exercise and diet vs. exercise only

##### 3.4.2.1. Body weight

**Figure 5.**
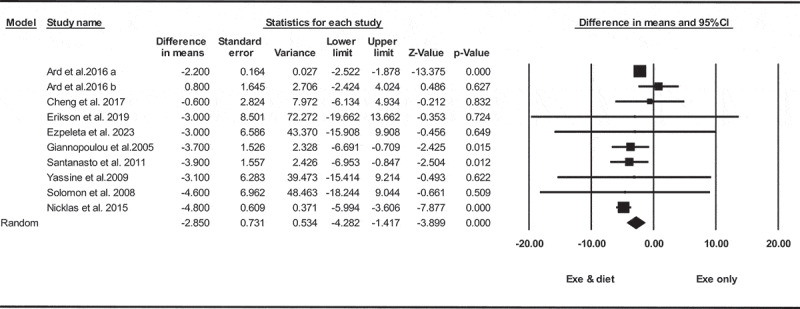
Forest plot of the effects of exercise and diet vs. exercise only on body weight. Data are reported as WMD (95% confidence limits). SMD, weighted mean difference.

Based on 10 interventions arms with 469 participants, exercise and diet significantly reduced in body weight [WMD = −2.85 kg (95% CI −4.28 to −1.41), *p* = 0.001], when compared with an exercise only group (Figure 5).

Subgroup analyses by health status revealed exercise and diet significantly reduced in body weight for adults with metabolic disease [WMD = −3.05 kg (95% CI −5.53 to −0.57), *p* = 0.010, five interventions], or individuals without metabolic disease [WMD = −2.78 kg (95% CI −4.66 to −0.89), *p* = 0.004, five interventions], compared with an exercise only group.

In addition, subgroup analyses by intervention duration revealed exercise and diet significantly reduced in body weight for long-term > 12 weeks [WMD = −2.78 kg (95% CI −4.38 to −1.18), *p* = 0.001, six interventions], but not for short-term interventions ≤12 weeks [WMD = −3.42 kg (95% CI −10.23 to 3.38), *p* = 0.320, four interventions], compared with an exercise only group. Due to the small number of studies, it was not possible to perform subgroup analysis based on BMI.

There was significant heterogeneity among included studies (I^2^ = 60.75%, *p* = 0.006). Visual interpretation of funnel plots and Egger’s test (*p* = 0.540) results also did not show publication bias. Sensitivity analysis performed by removing individual studies showed that the significance of the results and the direction of the results did not change.

##### 3.4.2.2. Liver fat

**Figure 6.**
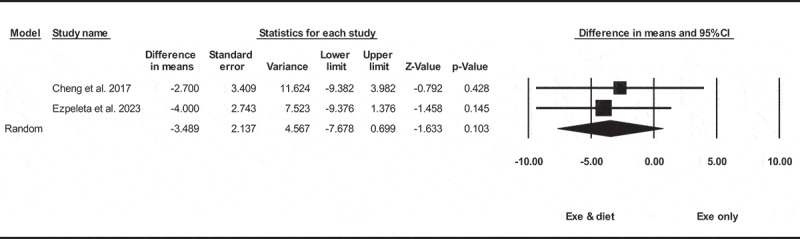
Forest plot of the effects of exercise and diet vs. exercise only on liver fat. Data are reported as WMD (95% confidence limits). WMD, weighted mean difference.

Based on two intervention arms with 90 participants, exercise and diet did not significantly change liver fat [WMD = −3.48% (95% CI −7.67 to 0.69), *p* = 0.100], when compared with an exercise only group (Figure 6). There was not significant heterogeneity among included studies (I^2^ = 0.00%, *p* = 0.760).

Due to the small number of studies, it was not possible to perform subgroup analysis based on health status, intervention duration, and BMI.

##### 3.4.2.3. VFA

**Figure 7.**
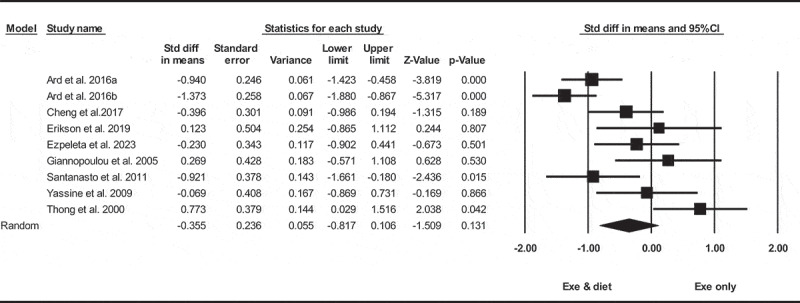
Forest plot of the effects of exercise and diet vs. exercise only on VFA. Data are reported as SMD (95% confidence limits). SMD, standardized mean difference.

Based on nine intervention arms with 368 participants, exercise and diet did not significantly reduce VFA [SMD = −0.35 (95% CI −0.81 to 0.10), *p* = 0.130], when compared with an exercise only group (Figure 7).

Subgroup analyses by health status revealed exercise and diet significantly reduced in VFA for adults with metabolic disease [SMD = −0.17 (95% CI −0.52 to 0.18), *p* = 0.340, four interventions], or without metabolic disease [SMD = −0.51 (95% CI −1.25 to 0.22), *p* = 0.170, five interventions], when compared with an exercise only group.

In addition, subgroup analyses by intervention duration revealed exercise and diet significantly reduced in VFA for long-term > 12 weeks [SMD = −0.72 (95% CI −1.22 to −0.22), *p* = 0.004, five interventions] with a moderate effect size, but not for short-term interventions ≤12 weeks [SMD = 0.14 (95% CI −0.32 to 0.60), *p* = 0.540, four interventions], when compared with an exercise only group. Due to the small number of studies, it was not possible to perform subgroup analysis based on BMI.

There was significant heterogeneity among included studies (I^2^ = 76.25%, *p* = 0.001). Visual interpretation of funnel plots and Egger’s test (*p* = 0.020) results also showed publication bias. Sensitivity analysis performed by removing individual studies revealed that removing the Thong et al. (2000), studies resulted in a change in the effect size and significance (SMD = −0.51, *p* = 0.010) respectively, while the direction of the results remained consistent.

Meta-regression determined whether or not body weight loss influenced the effects of exercise and diet on VFA, in which significant correlation was found (coefficient: −0.15; 95% CI, −0.29 to −0.01; *p* = 0.030). This result suggested that there was the significant moderating effect of body weight loss.

##### 3.4.2.4. IMTG

Based on three intervention arms with 165 participants, exercise and diet did not significantly change IMTG [SMD = −0.25 (95% CI −0.56 to 0.05), *p* = 0.100], when compared with an exercise only group (Supplementary Figure S2). There was not significant heterogeneity among included studies (I^2^ = 0.00%, *p* = 0.610). Visual interpretation of funnel plots and Egger’s test (*p* = 0.930) results also did not show publication bias. Sensitivity analysis performed by removing individual studies showed that the significance of the results and direction of the results did not change.

Meta-regression determined whether or not body weight loss influenced the effects of exercise and diet on IMTG, in which no significant correlation was found (coefficient: 0.26; 95% CI, −0.06 to 0.58; *p* = 0.110). This result suggested that there was no significant moderating effect of weight loss.

### 3.5. Quality assessment

The methodological quality of individual studies was evaluated using the PEDro tool with scores ranging from 6–8 out of a maximum of 9 points. Five studies had a score of 8, six studies had scores of 7, and six studies scored 6. Most of the studies received lower scores due to three evaluation criteria (concealed allocation, blinding of all assessors, and outcome measures assessed in 85% of participants). The details of the quality of studies are provided in Supplementary Table S1.

## 4. Discussion

The objective of this research was to investigate the effects of diet-only, and combined exercise with dietary interventions versus exercise alone on ectopic fat reduction, and weight loss in adults with overweight or obesity.

Our results confirm that both caloric restriction and exercise training effectively reduce body weight. Direct comparisons between caloric restriction alone and exercise alone revealed that a low-calorie diet results in significantly greater weight loss. This is consistent with previous research suggesting that energy-restricted diets are often more effective for weight loss than exercise alone [31].

Supporting evidence from numerous studies in both men and women also indicates greater average weight loss with dietary interventions compared to exercise-only protocols [23,40,58–60,64,65,67,69–71]. For individuals with medical conditions, such as arthritis or cardiovascular disease, a low-calorie diet may be particularly beneficial, as it allows for controlled caloric intake without the need for potentially strenuous physical activity [76,77]. Additionally, dietary changes can reduce systemic inflammation and improve gastrointestinal health, factors that are especially important for individuals with related comorbidities [78]. While it remains difficult to isolate the relative effects of different diets and exercise doses in a single study, evidence suggests that most diets are effective if adhered to, regardless of macronutrient composition [79,80]. Long-term adherence is critical in any dietary or exercise intervention. For individuals with chronic conditions, psychological support, and sustained behavior change strategies (such as cognitive-behavioral therapy or motivational interviewing) are key to maintaining progress beyond the initial phases of weight loss.

Subgroup analyses demonstrated that both exercise-only and combined exercise-diet interventions significantly reduce VAT in overweight and obese adults. However, combined interventions were more effective in trials exceeding 12 weeks. Previous meta-analytic findings suggest that exercise alone can reduce VAT by approximately 6.1%, even in the absence of significant weight loss [81], whereas dietary interventions alone appear less effective. Notably, sex differences have been observed, with men exhibiting greater VAT reductions following exercise compared to women [23]. This may reflect biological factors such as hormonal influences estrogen promotes visceral fat accumulation in women [82,83], while androgens enhance lipolysis in men [84] as well as behavioral tendencies, with men reportedly engaging in more physical activity outside of study protocols [85]. This hormonal difference highlights the importance of tailoring interventions according to sex, potentially incorporating personalized training and dietary strategies that address these biological variances. Intervention characteristics such as exercise intensity and participant age also influence VAT reduction. High-intensity protocols have been shown to be more effective [86,87], while younger participants tend to demonstrate better adherence and understanding of exercise techniques [39]. However, the present study was unable to perform subgroup analyses based on sex, age, or exercise intensity due to limited reporting in the included studies. This limitation may partially explain why combined interventions did not significantly outperform exercise alone in shorter interventions ( < 12 weeks).

Meta-regression analysis revealed that weight loss significantly moderated the relationship between combined interventions and VAT reduction. This may be attributed to improved systemic insulin sensitivity and enhanced lipid oxidation capacity, both established outcomes of negative energy balance [88]. In the absence of pharmacological or surgical treatments for NAFLD, lifestyle modification remains the cornerstone of clinical management [89–91]. A weight reduction of 5–7% is considered clinically meaningful for reducing hepatic fat content [92,93]. However, the role of dietary macronutrient composition, such as the impact of higher fat or low-carbohydrate diets on liver fat, requires further investigation. Due to the small number of studies, we could not evaluate the moderating role of weight loss on hepatic fat reduction in this analysis. Given the variability in dietary composition, intervention duration, and participant characteristics, future comparative studies are warranted to evaluate effective strategies for reducing hepatic steatosis.

To date, no specific nutritional strategy has proven superior in reducing NAFLD [94,95]. Exercise contributes to liver fat reduction by enhancing calorie expenditure, increasing fatty acid oxidation, improving insulin sensitivity, and reducing systemic inflammation [22,96–98]. Exercise also improves lipid metabolism by reducing triglycerides and increasing HDL cholesterol [99], thereby decreasing the pool of lipids available for hepatic storage.

Intramuscular fat (IMF) is another ectopic fat depot associated with metabolic dysfunction and reduced muscle strength, both of which contribute to chronic disease risk [8,100]. Investigating how diet and exercise influence IMF is therefore clinically important. Some studies have found that calorie restriction may increase IMF [101,102], while others, such as Larson-Meyer et al., found no change [103]. Exercise especially aerobic, resistance, or combined modalities appears to reduce IMF [104]. Moreover, training on a low-carbohydrate, high-fat diet may enhance IMF utilization as an energy source, increasing fat oxidation during exercise [105]. This could explain why IMF levels were similar in both the combined and exercise-only groups in our study, as IMF might have been utilized as fuel during high-intensity activity, particularly in trained individuals [8]. Further research is needed to explore how dietary strategies, such as intermittent fasting or protein timing, could affect IMF metabolism in conjunction with exercise.

Optimizing adherence is critical for successful weight and fat loss interventions [106]. Most weight loss occurs within the first 6 months of a lifestyle program [16], yet the interventions included in this meta-analysis typically lasted only 12 weeks. The short duration may have limited the magnitude of observed changes, particularly in liver fat and IMF. Future interventions should consider longer follow-up periods to assess the sustainability of ectopic fat reduction and the maintenance of metabolic improvements.

A key challenge in the field is the lack of studies with sufficient methodological rigor to enable robust statistical comparisons. To enhance the quality of future studies, researchers should consider more rigorous methodologies, including randomized controlled trials with larger sample sizes and longer durations. Future research should investigate the independent and combined effects of exercise parameters, including volume, intensity, frequency, and duration on ectopic fat reduction. Additionally, more work is needed to compare the effectiveness of aerobic versus resistance training for promoting favorable metabolic adaptations [107]. Although reductions in VAT and other ectopic depots are promising, the evidence remains limited due to small sample sizes and a low number of eligible studies. Accordingly, findings should be interpreted with caution.

One limitation of this meta-analysis is the small sample size of participants in the 2–3 intervention groups for liver fat and IMTG. This limited sample size may have led to insufficient statistical power to detect significant changes in liver fat and IMTG with diet-only or combined exercise and dietary interventions versus exercise alone. While the comparison between the diet-only and exercise-only groups showed a non-significant trend, the lack of power may have prevented the detection of smaller yet clinically important differences. Another concern is the high level of heterogeneity for IMTG among the included studies assessing the effects of diet-only in comparison to exercise alone. This indicates that the studies varied significantly in their methodologies, populations, and other factors, potentially impacting the generalizability of the results. The substantial heterogeneity also hinders the ability to draw definitive conclusions across all studies. Finally, the exploratory nature of the analysis and the limited number of studies on liver fat and IMTG outcomes suggest caution in interpreting these findings. The results may not be applicable to larger or more diverse populations. Further research with larger sample sizes and more consistent study designs is needed to validate these findings.

## 5. Conclusion

Although not the primary outcome, our analysis found that combined exercise and dietary interventions resulted in significantly greater weight loss compared to exercise alone. Importantly, VAT reductions in long-term interventions were mediated by weight loss. No significant changes in hepatic fat or IMTG were observed. Future studies should explore the underlying mechanisms and identify targeted strategies that effectively reduce ectopic fat particularly VAT, hepatic fat, and IMF independent of weight loss.

## 6. Practical applications

Combining exercise with dietary interventions particularly caloric restriction should be prioritized in clinical and fitness settings to achieve greater reductions in VAT and body weight, especially in programs lasting longer than 12 weeks. For individuals with limited ability to engage in physical activity due to medical conditions, dietary modifications offer a practical alternative to support fat loss. High-intensity exercise may enhance VAT reduction, and interventions should include strategies to improve adherence over time, such as behavior support and individualized planning. Moreover, personalized approaches considering individual characteristics such as sex, age, and comorbidities could improve the effectiveness of these interventions. Monitoring changes in ectopic fat, not just total body weight, can provide better insight into metabolic health improvements.

## Supplementary Material

Supplemental Material
