# Supplementary material for: Combined exercise training and dietary interventions versus independent effect of exercise on ectopic fat in individuals with overweight and obesity: a systematic review, meta‐analysis, and meta-regression
Source: J Int Soc Sports Nutr. 2025 Jul 6;22(1):2528534. doi: 10.1080/15502783.2025.2528534 (PMC12239108; doi:10.1080/15502783.2025.2528534)
Supplement: Supplemental Material [file RSSN_A_2528534_SM9822.docx]

|  | | | | | | | | | | | | |
| --- | --- | --- | --- | --- | --- | --- | --- | --- | --- | --- | --- | --- |
| **Supplementary Table 1.** Risk of bias assessment (PEDro scale) | | | | | | | | | | |  |  |
| **Authors and Year of Publication** | **Criteria 1** | **Criteria 2** | **Criteria 3** | **Criteria 4** | **Criteria 5** | **Criteria 6** | **Criteria 7** | **Criteria 8** | **Criteria 9** | **Total** |  |  |
| Ard et al. (2018) [58] | 🗸 | 🗸 | 🗴 | 🗸 | 🗸 | 🗴 | 🗸 | 🗸 | 🗸 | 7 |  |  |
| Cai et al. (2021) [59] | 🗸 | 🗸 | 🗴 | 🗸 | 🗸 | 🗸 | 🗸 | 🗸 | 🗸 | 8 |  |  |
| Cheng et al. (2017) [62] | 🗸 | 🗸 | 🗴 | 🗸 | 🗸 | 🗸 | 🗸 | 🗸 | 🗸 | 8 |  |  |
| Croci et al. (2016) [72] | 🗸 | 🗸 | 🗸 | 🗸 | 🗴 | 🗴 | 🗸 | 🗸 | 🗸 | 7 |  |  |
| Dube et al. (2011) [40] | 🗸 | 🗸 | 🗴 | 🗸 | 🗴 | 🗴 | 🗸 | 🗸 | 🗸 | 6 |  |  |
| Erikson et al. (2019) [57] | 🗸 | 🗸 | 🗴 | 🗸 | 🗴 | 🗸 | 🗸 | 🗸 | 🗸 | 7 |  |  |
| Ezpeleta et al. (2023) [105] | 🗸 | 🗸 | 🗴 | 🗸 | 🗸 | 🗴 | 🗴 | 🗸 | 🗸 | 6 |  |  |
| Giannopoulou et al. (2005) [106] | 🗸 | 🗸 | 🗴 | 🗸 | 🗸 | 🗸 | 🗸 | 🗸 | 🗸 | 8 |  |  |
| Murphy et al. (2012) [107] | 🗸 | 🗸 | 🗴 | 🗸 | 🗴 | 🗸 | 🗴 | 🗸 | 🗸 | 6 |  |  |
| Nicklas et al. (2015) [66] | 🗸 | 🗸 | 🗴 | 🗸 | 🗸 | 🗴 | 🗸 | 🗸 | 🗸 | 7 |  |  |
| Racette et al. (2006) [108] | 🗸 | 🗸 | 🗴 | 🗸 | 🗴 | 🗸 | 🗴 | 🗸 | 🗸 | 6 |  |  |
| Ross et al. (2000) [27] | 🗸 | 🗸 | 🗴 | 🗴 | 🗴 | 🗸 | 🗸 | 🗸 | 🗸 | 6 |  |  |
| Santanasto et al. (2011) [61] | 🗸 | 🗸 | 🗴 | 🗸 | 🗸 | 🗴 | 🗸 | 🗸 | 🗸 | 7 |  |  |
| So et al. (2012) [109] | 🗸 | 🗸 | 🗴 | 🗸 | 🗴 | 🗸 | 🗴 | 🗸 | 🗸 | 6 |  |  |
| Solomon et al. (2008) [110] | 🗸 | 🗸 | 🗴 | 🗸 | 🗴 | 🗸 | 🗴 | 🗸 | 🗸 | 6 |  |  |
| Thong et al. (2000) [70] | 🗸 | 🗸 | 🗴 | 🗸 | 🗸 | 🗸 | 🗸 | 🗸 | 🗸 | 8 |  |  |
| Yassine et al. (2009) [63] | 🗸 | 🗸 | 🗴 | 🗸 | 🗴 | 🗸 | 🗸 | 🗸 | 🗸 | 7 |  |  |

Supplementary Figure 1. Forest plot of the effects of diet only vs. exercise only on IMTG. Data are reported as SMD (95% confidence limits). SMD, standardized mean difference

Supplementary Figure 2. Forest plot of the effects of exercise and diet vs. exercise only on IMTG. Data are reported as SMD (95% confidence limits). SMD, standardized mean difference
